# Hybrid RF-Si Xerogels: A Cost-Effective Proposal for Insulator Materials

**DOI:** 10.3390/ma15010265

**Published:** 2021-12-30

**Authors:** Samantha L. Flores-López, Sara F. Villanueva, Natalia Rey-Raap, Ana Arenillas

**Affiliations:** 1 Instituto de Ciencia y Tecnología del Carbono, INCAR-CSIC, Francisco Pintado Fe, 26., 33011 Oviedo, Spain; samantha.f@incar.csic.es (S.L.F.-L.); s.villanueva@incar.csic.es (S.F.V.); 2 Department of Physical and Analytical Chemistry, Oviedo University-CINN-CSIC, 33006 Oviedo, Spain

**Keywords:** hybrid xerogels, thermal insulation, cost-effective process, microwave heating

## Abstract

Hybrid xerogels RF/Si were synthesized by controlling the chemical variables involved in the polymerization process (i.e., molar ratios, dilution ratio, catalysts, etc.) and evaluated as insulator materials. Higher insulating performances were recorded for these hybrids compared with their counterparts made from only one of their components (i.e., RF or Si xerogels with similar porous characteristics). The analysis of chemical and structural features correlated with heat transfer methods was useful in understanding the sum of contributions involved in the thermal conductivity of RF/Si xerogels. Variables such as roughness and tortuosity can be used to improve the performance of xerogels from a different perspective. In this way, thermal conductivities of 25 mW/mK were achieved without lengthy process steps or special drying methods. Knowledge of material design and the use of microwave heating during the synthesis allowed us to approach a simple and cost-effective process. These results suggest that the hybrid materials developed in this work are a good starting point for the future of the massive production of insulation materials.

## 1. Introduction

The global situation regarding climate change and the depletion of resources used as the main source of energy has brought as a consequence new concepts not only in the generation but also in the consumption of energy. In this regard, a major challenge today is to make the ways of harnessing energy more efficient. It is at this point where thermal insulating materials become the key to energy conservation [1,2,3] by preventing heat gain or loss in any kind of system. The versatility required for these materials is as wide as the number of situations and environments in which they can be used. To meet such demand, materials of different nature have been tested as thermal insulators and depending on their performance are classified as insulators or superinsulators. A material is considered an insulator if its thermal conductivity (λ) is lower than 100 mW/mK, although materials with conductivities between 30–50 mW/mK are most commonly used in practice for insulation applications. On the other hand, superinsulators are the best performing materials with values below air conductivity (i.e., 25 mW/mK) [4,5,6].

The thermal properties of a material are the sum of its ability to transfer heat by convection, radiation and conduction, and each phenomenon occurring during heat transfer is directly connected with its composition and structural properties [1,7]. Convection requires a moving phase, so when gas transport limitation occurs, convection is not considered [8]. On the other hand, radiation is related to the ability for IR absorption [1,9] and therefore is associated with the chemical bonds present in the material and their chemical nature. Conduction involves both the gas and the solid phases present in the material, so it can be related to the properties of the porous and solid matrix [2,7,10,11]. 

On this basis, aerogels are considered a good option for insulation since their properties as non-ordered structures, higher porosity (i.e., more than 90%) and low weight [1,6,9,12] are able to neutralize or nullify the methods of heat transfer [7,8,13]. Aerogels are able to perform as superinsulators, and among the materials that achieve this characteristic, they are considered the most promising ones. Silica aerogels have the most extended use as superinsulators since their porous network is formed mainly by narrow pores (i.e., micro and mesopores), and they have a high porosity [1,3,14]. The small pore sizes of these aerogels allow nullification of the convection and gas conductivity, so only radiation and solid conductivity contribute to the heat transfer process [8,15]. These materials reach thermal conductivities around 10–20 mW/mK and have a wide variety of applications [9,13]. Nevertheless, their long and multi-step synthesis process, high production costs, poor mechanical properties and health issues (i.e., irritant to the eyes, skin, respiratory tract, and digestive system) limit their practical applications and mass production [1,6,16,17,18]. Another type of aerogels that have also attracted attention are those based on resorcinol–formaldehyde (RF). Based on the low conductivity of a nonporous RF material, Pekala and co-workers modulated the porous properties of the RF aerogels to achieve good insulators. Their work focused on pore size reduction while maintaining high porosity, considering it the most effective strategy for reducing thermal conductivity. Through the use of supercritical drying, RF aerogels with densities around 0.05–0.3 g/cm^3^ and competitive λ values as low as 10 mW/mK were obtained [7,9]. On the other hand, Rey-Raap et al. [19] modified the synthesis conditions to eliminate this expensive drying method by producing RF xerogels of low density and small pore size, equaling the insulating capacity of expanded polystyrene (i.e., 34 mW/mK). Compared with RF aerogels, the proposed synthesis methodology allows a reduction in the costs and time required for production; however, the relatively high thermal conductivities are an opportunity for improvement. Finally, a third and more novel version of insulators has been studied: hybrid aerogels. These materials are derived from the well-known silica gels, and they address the problem of poor mechanical properties by the addition of organic components (e.g., RF). Combining the advantages of their dual nature, these materials offer a major versatility in shapes and sizes (i.e., films, monoliths, etc.) and a wider range of interesting properties in the insulation field (e.g., flexibility, transparency or adsorptive characteristics) [16,20,21,22]. However, to maintain a remarkable insulating performance in these hybrid gels, the optimal characteristics (i.e., structure and low density) are reached by supercritical drying. To avoid this complicated and time-consuming procedure, strategies for ambient pressure drying have been explored. Li et al. [10] and Yun et al. [23] added methyltrimethoxysilane (MTMS) as a silica precursor in silica and hybrid (RF/Si) gels, respectively, to provide hydrophobic character to the samples and facilitate the drying step. Thermal conductivities of 26 and 38 mW/mK were achieved for silica and hybrid xerogels, respectively. The use of MTMS improve the mechanical resistance of the xerogels avoided shrinkage during drying, but did not favor the insulating performance [18,23]. Thus, new strategies focusing on the role of chemical and porous properties should be adopted to optimize the insulating performance of hybrid xerogels. Additionally, the low-cost production technology for mass commercialization of gel-based insulation products with improved isolation performance is still a current challenge [1,3,11,24].

On the other hand, silica precursors that include amino groups into their structure have been employed to promote polymerization between RF and silica species (e.g., 3-aminopropyltriethoxysilane (APTES) and 3-[2-aminoethylamino]propyl] trimethoxysilane (AEAPTMES)), reaching small gelling times for hybrid synthesis [20,21]. Moreover, a previous study has evaluated the use AEAPTMES as a catalyst, which when coupled with microwave heating resulted in a fast process, allowing the control of the porous properties [25]. Therefore, the use of AEAPTMES becomes a promising synthesis option for applying and for evaluating the performance of the obtained materials in many applications.

This work proposes a cost-effective synthesis route for obtaining hybrid xerogels with suitable characteristics to perform as insulators. To achieve this goal, hybrid RF/Si materials were synthesized through a sol–gel process assisted by microwave (MW) heating, followed by ambient pressure drying and using AEAPTMES as a catalyst. These strategies reduce the time required for the synthesis and avoid the mentioned problems in the drying step. Conventionally synthesized hybrid materials (i.e., by conventional heating) were included to demonstrate that there are no additional advantages in comparison with those synthesized by microwave heating. In addition, different molar ratios of reagents were employed to vary the Si content and structural features of the hybrid materials. Then, the optimization of the synthesis conditions was performed through the analysis of the different contributions to the heat transfer and their correlation with the material properties. Finally, a competitive insulating performance was achieved for the RF/Si materials. 

## 2. Experimental

### 2.1. Reagents

Reagents employed for the synthesis were resorcinol (R, Indspec 99%), formaldehyde (F, Merck37%), tetraethylorthosilicate (TEOS, Chem-Lab, 99%), deionized water, ethanol (EtOH, J.T. Baker, 96%), 3-[2-aminoethylamino]propyl]trimethoxysilane (AEAPTMES, Sigma Aldrich 97%), ammonia 2M (Acros Organics, 25%), ammonium fluoride 0.05 M (HN_4_F, Sigma-Aldrich 98%) and NaOH (VWR, 99%). 

### 2.2. Synthesis of Hybrid Xerogels

The synthesis of the hybrid xerogels involved the sol-gel process. The preparation of the precursor mixture (1 L) started by dissolving R in the solvent (i.e., water and/or EtOH). Then the corresponding quantities of TEOS and F were incorporated, and the mixture remained under magnetic stirring for 2 h. Once the solution became homogenous, AEAPTMES was added, maintaining the agitation for one minute. The mixture was then heated at 60 °C for gelation, curing and drying. In order to compare different synthesis methods, two heating mechanisms were employed:

(a) Convection heating. The synthesis process was carried out in an electric oven for 72 h in a closed vessel to promote the gelation and aging step.
(b) Microwave heating. The samples were under MW heating for 5 h. Initially, the reaction container was maintained closed for 3 h to promote the gelation and aging step. Then, the container was opened and kept under MW heating for 2 h to initiate the drying stage.

Regarding the synthesis heating method, all samples were then completely dried in the electric oven. The reaction vessels (opened) were heated at 60 °C until constant weight. 

The concentration of each reagent was selected based on previous publications [25,26,27,28] in order to obtain hybrid xerogels with different compositions, a wide range of envelope density and small pore size. For this purpose, the commonly used molar ratios of the reagents were fixed. The R/TEOS ratio was employed to vary the silica content on the samples from 70% to 10% (i.e., R/TEOS ratio from 0.1 to 6). On the other hand, the R/F and EtOH/TEOS molar ratios and the dilution ratio (D, molar ratio between the total solvent and reactants) were systematically adjusted to obtain hybrid xerogels with envelope densities between 0.35 and 0.85 g/cm^3^ and a pore size smaller or equal to 100 nm. Therefore, the R/F, EtOH/TEOS and D were set within the ranges 0.1–0.5, 0–5 and 5.6–12.9, respectively (detailed molar ratios in Table S1, Supplementary Materials). The concentration of AEAPTMES was fixed at 5% (vol.) of the total amount of TEOS, based on our previous work [25].

Non-hybrid RF and Si xerogels were prepared for comparison. In terms of composition, these materials corresponded to the limits of 0% and 100% of Si content, respectively. These non-hybrid xerogels were synthesized selecting the appropriate concentration of reagents (based on our previous work [14,19,29]) to achieve envelope densities and pore sizes comparable with those obtained for the hybrid RF/Si xerogels. Such samples were entirely synthesized by MW heating following the methodology described in our previous publications [28,30,31]. A brief description of the synthesis conditions can be found in Supplementary Materials. 

The samples were labeled as follows: Si, RF or RF/Si for silica, resorcinol–formaldehyde and hybrid xerogels, respectively, followed by the value of their envelope density in g/cm^3^. The only sample with a high silica content also included these data, being labeled as RF/Si-0.50–70%. In the corresponding cases, “C” is added, indicating the use of conventional heating for synthesis.

### 2.3. Textural and Chemical Characterization

Samples previously dried under vacuum at 200 °C overnight were used for textural characterization. The pore network was analyzed for all samples. Helium density (*ρ_He_*) was measured by He pycnometry using an AccuPyc 1330 from Micromeritics. Envelope density (*ρ_env_*) was determined with the Envelop density analyzer GeoPyc1360 (Micromeritics). Porosity (*P*) and total pore volume (*V_T_*) were calculated from both density values, using the following equations:

(1) $$P=\left(1-\frac{\rho_{Env}}{\rho_{He}}\right)100$$

(2) $$V_{T}=\left(\frac{1}{\rho_{Env}}-\frac{1}{\rho_{He}}\right)$$

N_2_ adsorption–desorption isotherms (at 77 K, Tristar II 3020 from Micromeritics) and mercury intrusion porosimetry (MIP, AutoPore IV from Micromeritics) were carried out to characterize the entire porous range. *V_T_* values calculated from Equation (2) were confirmed with pore volumes obtained from N_2_ isotherms and MIP. The mean pore sizes (*d_p_*) were determined by the Washburn equation (MIP) for RF and hybrid samples (i.e., RF/Si) and by applying the BHJ model to the N_2_ isotherms in the case of Si samples. The specific surface area (*S_BET_*) was calculated with the BET model in all cases.

A ThermoNicolet-6700 FT-IR spectrometer equipped with a DTGS-KBr detector was used to determine the surface chemical groups of the xerogels. FT-IR spectra (400–4000 cm^−1^) were obtained from KBr pellets (dilution 1:50) prepared and dried under vacuum overnight at 200 °C. For specific samples, the solid network was also analyzed. The microstructure of the different samples (previously metallized) was examined using a scanning electron microscope (SEM, Quanta FEG 650). The tortuosity factor (τ) was calculated from MIP, employing MicroActive and PoreXpert software (methodology explained in detail in reference [32]).

### 2.4. Thermal Conductivity Analysis

The thermal conductivity was measured based on the transient hot plane method with the thermal conductivity analyzer TPS 2200 Hot Disk. The isotropic module for low-density materials, a holder sample (capacity 250 mL) and the Kapton insulated sensor 8563 (radius: 9.868 mm) were employed for the analysis. HotDisk equipment was stabilized previously to each measurement. As the user manual recommends, power heat and analysis time were carefully adjusted on each sample to obtain adequate values of transient (i.e., 3–5), probing depth (i.e., around 13 mm) and total characterization time (i.e., 0.35–1). Two commercial solid materials were used to calibrate the sensor and establish the methodology of analysis: Foamglass^®^ (insulator) and CASSPIR^®^ (superinsulator). Moreover, a granular commercial sample, Lumira^®^ (superinsulator), was also used as a reference to verify the measurements. Measurement conditions and the results obtained for these reference samples are presented in Table 1. These results match perfectly with the technical specifications reported for each material, demonstrating that the equipment was successfully calibrated within the range of low thermal conductivity values. 

**Figure 1:**
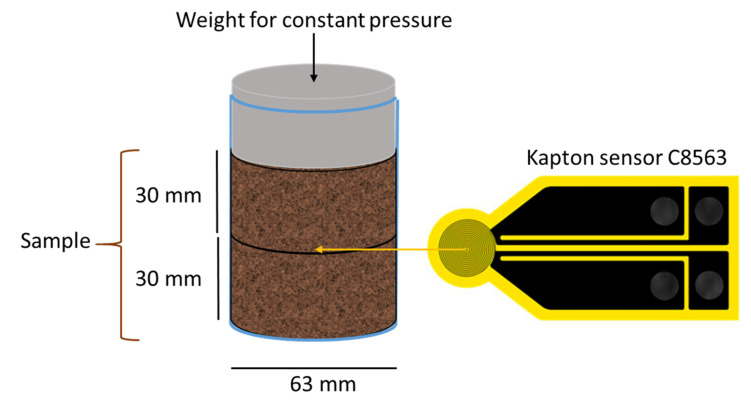
Configuration of the system for thermal conductivity measurements.

The thermal conductivity of the xerogels synthesized in this study were measured following this verified protocol. Measurements were performed at room temperature, using previously degassed samples (200 °C, 0.1 mbar, 12 h) and two different particle sizes, 1–2 mm and 0.5–0.212 mm. Both conditions minimize the thermal conductivity value [19]. The HotDisk probe was placed in the middle of the holder, leaving two cylindrical sample sections of equal dimensions (63 mm diameter and 30 mm height) on each side (see Figure 1). The power heat ranged from 0.02 to 0.04 (this value increases with density), while the analysis time was kept at 320 s. Because our samples were granular, a uniform and constant pressure was ensured in each analysis. All samples were tested in triplicate to obtain an average value of the thermal conductivity (λ).

To understand the phenomena involved in the thermal conductivity occurring in the xerogels, the contribution of each transfer heat method (convection, radiation and conduction) was calculated. Convection was not taken into account due to the small pore structure of the materials. Radiation implies heat transfer between two media, caused by the emission of electromagnetic radiation through the surface. Pekala argued that materials with good IR adsorption should be better insulators than those with poor IR adsorption. He showed that, due to the presence of carbon, most organic polymers are good IR absorbers, e.g., RF materials have an extinction coefficient (*K_e_*) of 50.1 m^2^/Kg [9,33]. On the other hand, silica aerogel shows lower absorption with a *K_e_* equal to 22.7 m^2^/Kg [9]. For porous materials, the use of Rossland diffusion approximation (Equation (3)) leads to satisfactory results in the calculation of radiation (*λ_r_*). Then, this equation was used to calculate *λ_r_*, which includes the refraction index (*n* = 1), the Stefan–Boltzmann constant (*σ* = 5.67 × 10^−8^ W/m^2^K^4^), the room temperature (*T* = 298 K), the envelope density (*ρ_env_*) and the IR adsorption capability of the materials expressed as their extinction coefficient (*K_e_*). For hybrid materials, this coefficient was estimated based on the individual *K_e_* values of the components (RF or Si) and the percentage composition [2,33].

(3) $$\lambda_{r}=\frac{16n^{2}\sigma T^{3}}{3K_{e}\rho_{env}}$$

On the other hand, the porous nature of the xerogels separates the contribution by conduction in gas and solid conductivity. Gas conductivity (*λ_g_*) refers to the heat transfer occurring between the gas molecules present in the porous matrix of the material. *λ_g_* was calculated by the following equation, which takes into account the contribution of porosity and pore size [7,10,33]:

(4) $$\lambda_{g}=\frac{\lambda_{g}{}^{o}µ}{1+2\beta K_{n}}$$

where $\lambda_{g}{}^{o}$ is the bulk gas conductivity (i.e., 26 mW/mK for air), *µ* the porosity fraction, *β* the efficiency of the energy transfer between gas molecules (i.e., 1.94), and *K_n_* the Knudsen number, defined as the ratio between the mean free path of gas molecules ($l_{mean}$, 72.2 nm at 1 atm, 30 °C for air) and the pore size (d_p_) [2,33].

From Equation (4), it can be inferred that conduction through the gas molecules within the pores decreases with the presence of small pore sizes. Small pore sizes lead to a higher probability of collisions with pore walls instead of other gas molecules, which increases the Knudsen number and hence decreases the gaseous thermal conductivity [2,9].

Finally, solid conductivity (*λ_s_*) depends on the contact area between the clusters, solid conduction path and interface thermal resistance. The *λ_s_* value of each material was calculated by the difference between *λ* and *λ_r_* + *λ_g_*.

## 3. Results and Discussion

The properties of the materials synthesized in this work are summarized in Table 2. As expected, the hybrids xerogels showed *ρ_env_* into a wide range between 0.36 and 0.88 cm^3^/g due to the control of the synthesis conditions. Since their *ρ_He_* were similar (1.41–1.56 cm^3^/g), the porosity varied between 76% and 43% according to *ρ_env_* (Equation (1)). Much like porosity, the total pore volume also increased by decreasing *ρ_env_* (Equation (2)), being over 1 g/cm^3^ for most of the samples. As expected from the synthesis design, hybrids with narrow pore sizes (less than 100 nm) were obtained with the aim of achieving low conductivity values [9,19]. For hybrid xerogels synthesized by microwave heating, the initial drying performed under microwave heating resulted in a slight collapse, and consequently less pore volume and smaller pore size. For this reason, in order to increase these properties, lower R/F or higher D were employed in the xerogels made by microwave heating (see Table S1). In addition, the quick gelation resulted in a mesoporous network with low microporosity (see Figure S1c,d, Supplementary Materials). Then, lower S_BET_ values were reported for samples synthesized by MW, although in all cases the area of the hybrid materials did not exceed 230 m^2^/g. Hybrid RF/Si-0.50–70% was a particular case. Its high silica content and the synthesis conditions used involved a high amount of catalyst present in the precursor mixture, which favored rapid polymerization. As a consequence, this sample presented a high *ρ_He_* and a macroporous structure. However, the rest of the parameters compared well with the other hybrid xerogels. From the data shown in Table 2, it can also be seen that the Si and RF xerogels were designed to match *ρ_env_* and *d_p_* of the hybrid xerogels for comparison. Si materials had higher *ρ_He_*, which led to higher *P* and *V_T_* in comparison with hybrids at the same *ρ_env_*. Moreover, the values of S_BET_ were around 550 m^2^/g, indicating the presence of a major microporosity characteristic of these polymeric structures (see Figure S2a) [15,29]. On the other hand, the RF xerogels perfectly fit all their porous properties in the ranges defined for the hybrid series, including their small S_BET_, close to those of RF/Si samples synthesized in MW (see Figure S2b,d). This similitude can be attributed to the low content of silica in the hybrid samples (i.e., 10 or 15%), which resulted in porous properties closer to those of the RF xerogels. 

**Figure 2:**
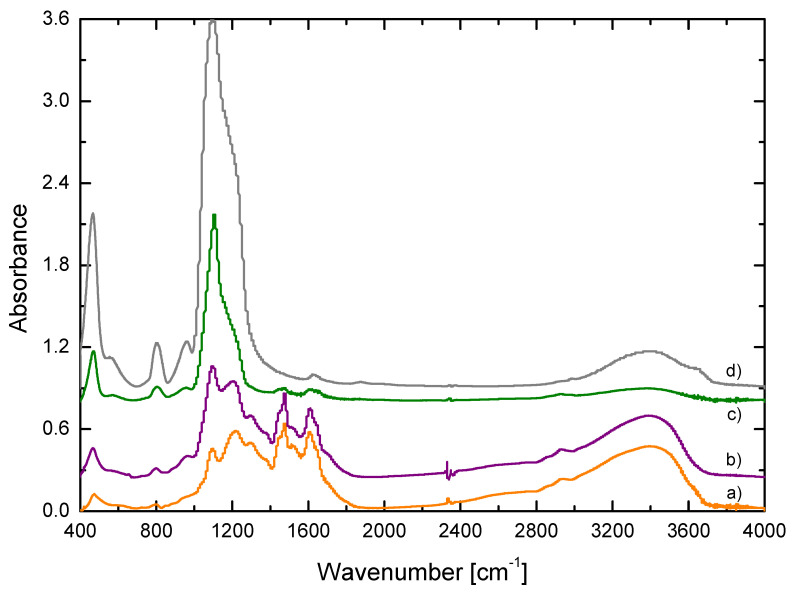
FT-IR spectra of selected xerogels with different composition: (a) RF-0.55, (b) RF/Si-0.55, (c) RF/Si-0.50–70% and (d) Si-0.50.

In addition, to corroborate their chemical composition, Figure 2 shows the representative FT-IR spectrum of each kind of material synthesized. The spectrum for RF xerogel (see Figure 2a) showed the characteristic bands for these organic materials, which can be assigned as follows: the methylene ether bridges C-O-C (474, 1090, 1217 and 1298 cm^−1^), aliphatic C-H bonds (1459 cm^−1^), aromatic C-H bonds (1511 cm^−1^), aromatic ring stretching vibrations C=C (1610 cm^−1^), C-H stretching vibrations (2930 cm^−1^) and -OH stretching vibrations (3000–3600 cm^−1^) [25,34,35]. On the other hand, bands in the Si spectrum (see Figure 2d) corresponded to those of a silica material synthesized from TEOS: the asymmetric stretching vibration and bending vibrations of Si-O-Si (470, 796 and 1090 cm^−1^), Si-OH bonds (970 and 3400 cm^−1^) and CH2 stretch (1600 cm^−1^) [20,25,28]. On its part, the hybrid samples with 15% and 70% of Si content (Figure 2b,c, respectively) showed the characteristic bands of both organic and inorganic contributions. Characteristic bands of Si bonds (i.e., 400–1100 cm^−1^) showed a higher intensity for RF/Si-0.50–70%, while the characteristic bands for the RF contribution (i.e., range 1400–2000 cm^−1^) were more intense in the sample RF/Si-0.55 (i.e., hybrid with low Si content).

To illustrate the role of different properties of xerogels on the total thermal conductivity, the contributions of chemical nature, porous structure and solid matrix were analyzed by the heat transfer method involved in each case.

### 3.1. Chemical Composition Contribution

**Figure 3:**
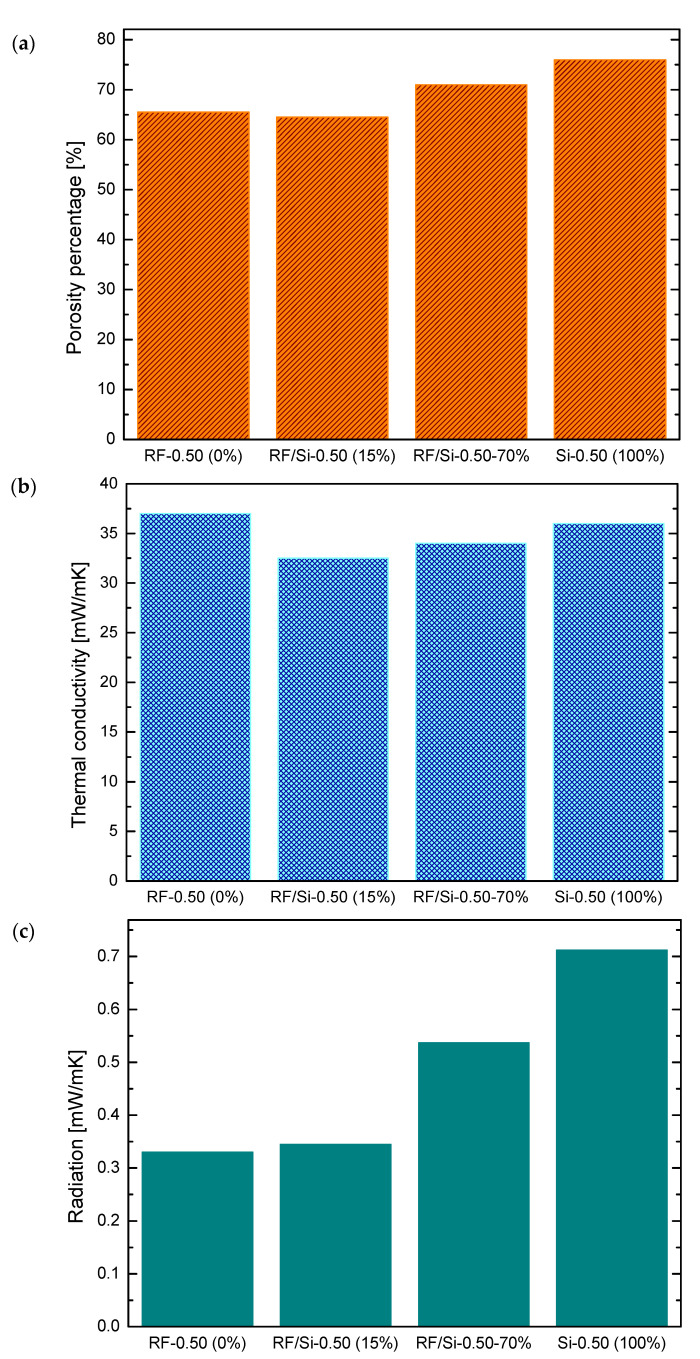
Porosity (**a**), thermal conductivity (**b**) and contribution of radiation (**c**) of xerogels with different Si content and similar envelope density (0.50 g/cm^3^).

To evaluate the effect of the chemical composition on the thermal properties, hybrid samples with different Si content and the same envelope density (i.e., 0.50 g/cm^3^) were compared. Figure 3 shows the porosity and thermal conductivity of these xerogels: RF-0.50 (0% of Si), hybrid RF/Si-0.50 (15% of Si), hybrid RF/Si-0.50–70% (70% of Si) and Si-0.50 (100% of Si). For RF and RF/Si with 15% Si percentage, the values reported in this section correspond to the average of samples RF-0.45, RF-0.55, and RF/Si-0.45, RF/Si-0.55, RF/Si-0.45-C, RF/Si-0.55-C, respectively.

At a same *ρ_env_*, the porosity increased with the Si content (Figure 3a). Some studies have reported that in comparison with RF aerogels, an increase in porosity in Si materials results in a lower thermal conductivity [7]. However, this behavior was not observed in the hybrid samples, as the material with the lowest porosity exhibited the lowest thermal conductivity (Figure 3b). This phenomenon was probably due to the chemical composition, which has a great impact on thermal conductivity due to its contribution to radiation. Therefore, to quantify the influence of this variable in our xerogels of different nature, radiation was calculated with Equation (3). 

The values of the radiation are shown in Figure 3c. Samples with higher Si content possessed the highest contribution by radiation to the thermal conductivity. Even if the porosity value of Si material was the highest between this series of samples, its radiation had a great impact on their total thermal conductivity [15]. Therefore, the contribution of the chemical composition through radiation was significant. To obtain a high insulating performance, it was necessary to combine the porous properties generated by the presence of Si and the low radiation capacity provided by the RF. Hence, both hybrid materials (i.e., RF/Si-0.50 and RF/Si-0.50–70%) had the lowest thermal conductivities (see Figure 3b). However, the hybrid with 70% of Si content did not achieve the lowest conductivity value due to its pore size of the order of microns, while the rest of the samples had pore sizes around 41nm. In consequence, the sample with the lowest Si content was the one with the optimum combination of properties to achieve the best insulating performance.

### 3.2. Porous Matrix Contribution

**Figure 4:**
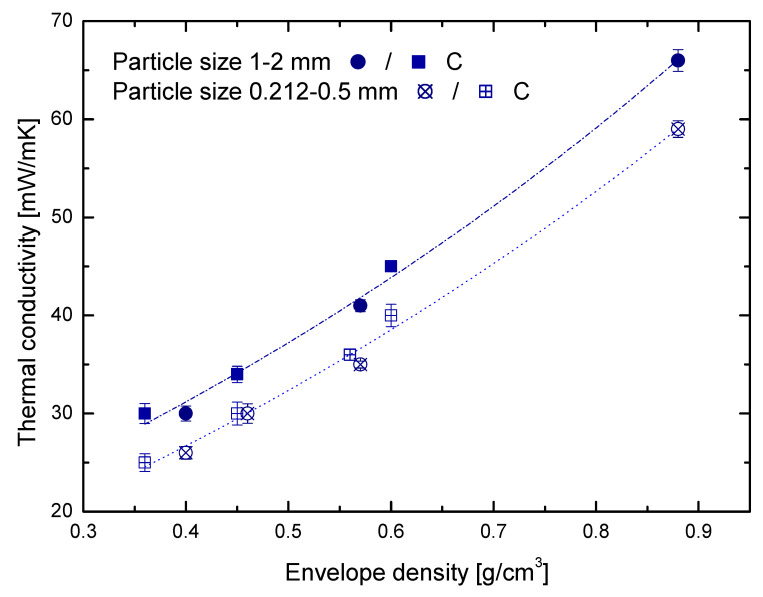
Thermal conductivity of hybrid xerogels versus envelope density measured at two different particle sizes (numerical values can be found in the Supplementary Materials, Table S3).

The contribution of the porous matrix was analyzed in a series of samples prepared from the optimized Si content (i.e., 10–15% Si). Figure 4 shows the thermal conductivity (λ) of these hybrid samples versus the envelope density (*ρ_env_*) measured at two different particle sizes. Regardless of the envelope density, the reduction in particle size from 1–2 nm to 0.212–0.5 mm significantly decreased the thermal conductivity (ca. 12%). These results are in agreement with those previously published for RF xerogels [19]. Additionally, it should be noted that the heating method used for the synthesis (i.e., conventional and microwave heating) had no particular effect on the thermal conductivity. Regardless of the heating method, samples with similar *ρ_env_* showed similar λ. These results guarantee an adequate synthesis process via microwave heating that, in comparison with the conventional one, allowed reducing the synthesis time to just a few hours. 

On the other hand, porous matrix contribution was intimately related to porous parameters such as density and pore size. In fact, it has been already observed that the insulation performance can be improved by decreasing the value of these parameters [9,12,19]. In agreement with the results in the literature, the thermal conductivity of the hybrid xerogels showed strong density dependence with an exponential behavior (Figure 4). For instance, a comparison between sample RF/Si-0.55-C and RF/Si-0.45-C, both with similar mean pore sizes (i.e., 43 and 42 nm, respectively), demonstrated a reduction of 16.66% in λ by the effect of the density (from 36 to 30 mW/mK). The same behavior with a further reduction in λ of 40.67% (from 59 to 35 mW/mK) was observed by comparing sample RF/Si-0.85 and RF/Si-0.55 (with pore sizes of 28 and 31 nm, respectively). However, similar λ values are observed by comparing samples with similar envelope density (RF/Si-0.55-C and RF/Si-0.55) but different pore sizes (43 and 31 nm, respectively). Therefore, it can be concluded that the pore size’s effect on the hybrid xerogels studied was much lower than that of the density. However, if pore size shows differences by an order of magnitude the reduction in thermal conductivity is noticeable [19]. In this case, the pore size of hybrid materials was minimized to favor the insulating performance. Then, the absence of the effect of this variable may be attributed to the small d_p_ values reported here. Nevertheless, pore size contributes to the heat transfer through the gas filling the empty spaces of the material (air for the material used in this work). In the case of RF/Si hybrid xerogels, heat transport thought convection was not taken into account due to the small pore size dimension and the absence of gas flow [3,8]. Then, pore size mainly impacted the gas conductivity of the materials. Using Equation (4), gas conductivity was calculated for each sample. 

**Figure 5:**
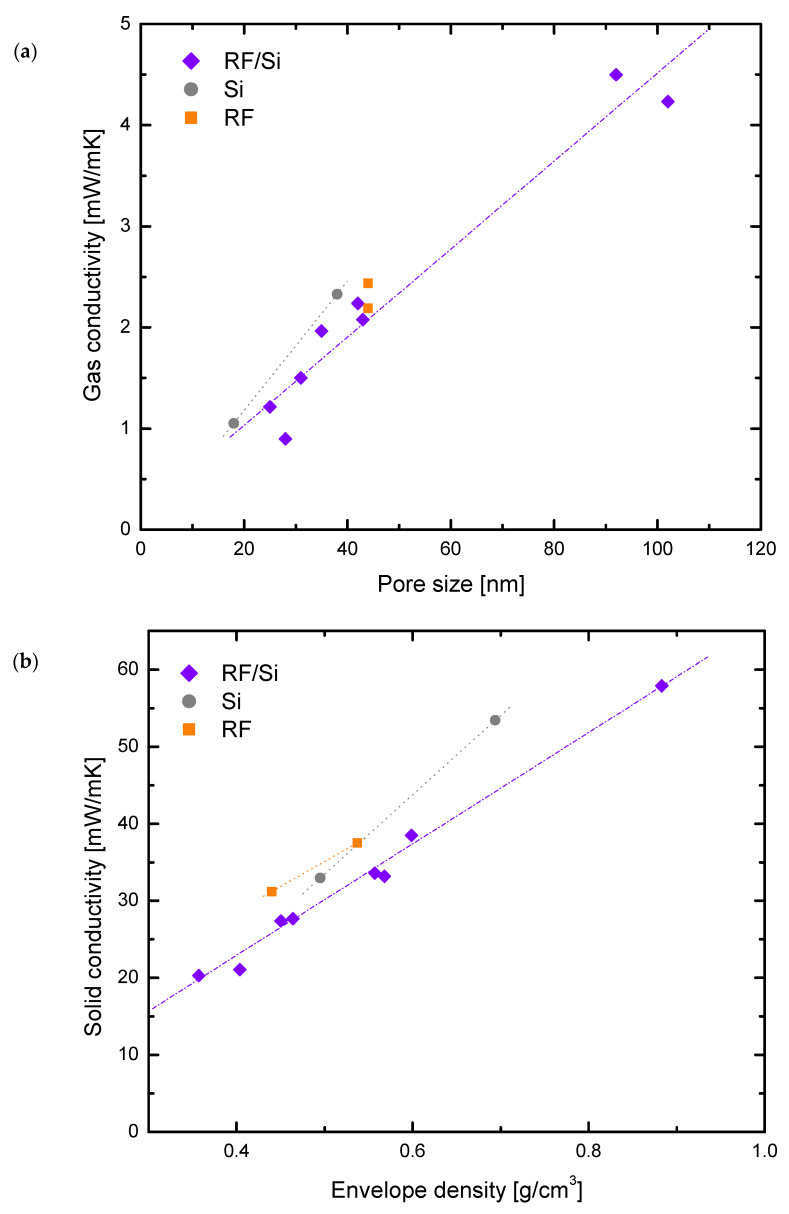
Contribution to the total thermal conductivity of two of the main heat transfer methods: gas conductivity (**a**) and solid conductivity (**b**).

Figure 5a shows the gas conductivity versus pore sizes for non-hybrids and the low Si content hybrid series. Hybrid and non-hybrid samples with pore sizes ranging from 20 to 45 nm had λ_g_ values below 3 mW/mK, while samples with larger pore sizes (ca. 92 and 102 nm) corresponded to higher gas conductivity (ca. 4.5). Moreover, sample RF/Si-0.50–70% (*d_p_* = 5395 nm) had a gas conductivity of 17.5 mW/mK, which corresponded to 51% of its total thermal conductivity (not included in Figure 5a). These values were in good agreement with the Knudsen effect present in each sample. Since 72.2 nm is the mean free path of the air, in samples with smaller pore sizes (i.e., *K_n_* > 1) the gas conductivity was almost nullified. On the other hand, when the pore size exceeded this value, the gas conductivity started to have a larger contribution, increasing accordingly [2,7].

It is worth noting that among the hybrids with low Si content, samples with the small *ρ_env_* (i.e., RF/Si-0.35-C and RF/Si-0.40) presented the lowest thermal conductivities (ca. 26 mW/mK), even if their pore sizes were the bigger ones (see Table 2). With pore sizes being close to the determinant value in the Knudsen effect (i.e., 72.2 nm in this case), they contributed to blocking or turning aside the conduction paths in the porous matrix, so the gas conductivity did not have a relevant impact, allowing their low density to contribute to their insulating performance. Then, even the pore size had its contribution on these materials; their modulated small pores avoided its appreciation in the thermal conductivity values. Thus, if the pore size had an irrelevant contribution, the advantage of the hybrid materials observed in Figure 3b must be attributed to the solid matrix contribution.

### 3.3. Solid Matrix Contribution 

**Figure 6:**
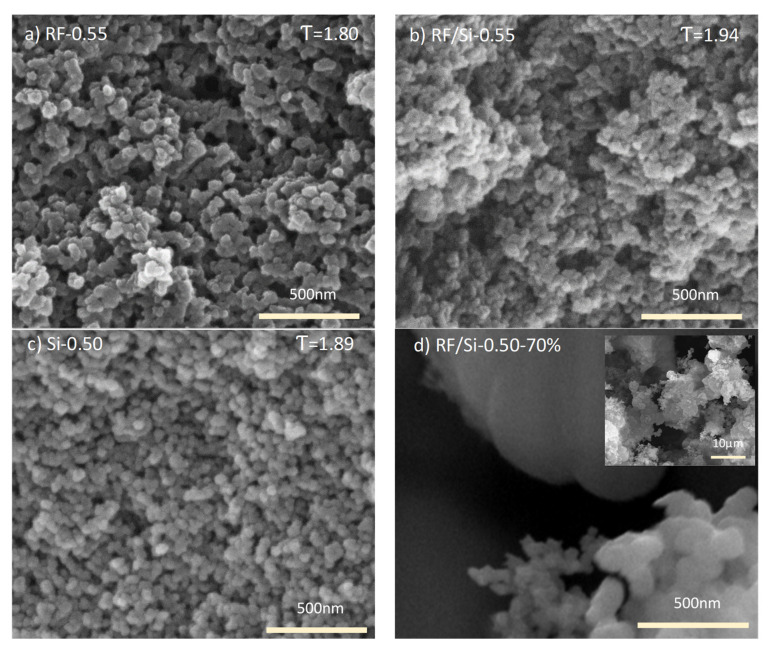
SEM micrographs of the different synthesized xerogels: (**a**) RF-0.55, (**b**) RF/Si-0.55, (**c**) Si-0.50 and (**d**) RF/Si-0.50–70%.

The solid conductivity (i.e., solid matrix contribution) was calculated by the difference between total thermal conductivity (experimentally measured) and the contribution of the radiation and gas conductivity determined in Section 3.1 and Section 3.2, respectively. The solid conductivity was calculated for both hybrid and non-hybrid xerogels, and their values are shown in Figure 5b. The tendency of solid conductivity for the three types of xerogels (RF, Si and RF/Si) was similar, i.e., decreases with the envelope density. However, slightly larger values were observed for RF and Si materials than for the hybrid xerogels. These values demonstrate that the solid matrix of the hybrid materials had adequate properties for reducing conductivity, contributing to improve the insulator performance. The solid conductivity is highly dependent on the microstructure, i.e., cluster size, necks, connections [2,33]. So, the morphology of the network of RF, Si and RF/Si materials was analyzed by SEM (see Figure 6).

Hybrid materials had smaller clusters than those obtained in RF samples [20,25] (see Figure 6, (a) RF-0.55 and (b) RF/Si-0.55). In comparison with RF xerogels (pH 4.5), the synthesis of hybrid xerogels was carried out at elevated pH values (ca. 7) due to the presence of amino groups in AEAPTMES. Accordingly, the formation of a large number of small clusters of RF species occurred quickly [26]. Moreover, in RF/Si samples with a low Si content, Si monomers were easily added to these clusters by AEAPTMES, since it acts as bonding between organic (RF) and inorganic (Si) species [23,25]. On the other hand, no difference in cluster size was appreciated by comparison of samples Si-0.50 and RF/Si-0.55 (see Figure 6b,c respectively). Even if different pH values were also registered for these samples, their similar cluster size could be attributed to the use of a basic catalyst of similar nature [14,29]. However, clusters in RF/Si-0.55 were less spherical and homogeneous than those of Si-0.50, affecting the photon scattering through the solid matrix. High heterogeneity and a large number of small clusters in the hybrid xerogel increased the interface thermal resistance and photon scattering, leading to a decrease in the solid thermal conductivity [10,33]. In the particular case of sample RF/Si-0.50–70%, synthesis conditions (i.e., pH around 9 and the high Si content) generated the most heterogeneous network with clusters of different sizes (see Figure 6d). This morphology was in good agreement with the lower value of solid conductivity of such sample of just 16 mW/mK. 

Material morphology is correlated with parameters such as fractal dimension and tortuosity factor [33,36,37], which are key parameters of transport properties. For this reason, the tortuosity factor (τ) was calculated (τ values inset in Figure 6) from PIM results. These values were affected by the mean free path of the sample (ca. 41 nm for the hybrid series with low Si content). In terms of the solid matrix, a high tortuosity value represents a more disordered structure, with dangling terminals or complex crosslinks that complicate the conduction paths. As a consequence of these characteristics, solid conductivity decreases [1,33,36,38]. In agreement with Figure 5b, Si-0.50 showed an intermediate τ, which means an intermediate λ_s_. By comparison with RF-0.55, RF material showed fused clusters—this characteristic increased the contact in the solid network, which resulted in a higher thermal conductivity. On the other hand, comparing Si with the hybrid material (RF/Si), the low τ in sample Si-0.50 could be attributed to a more ordered structure. 

**Figure 7:**
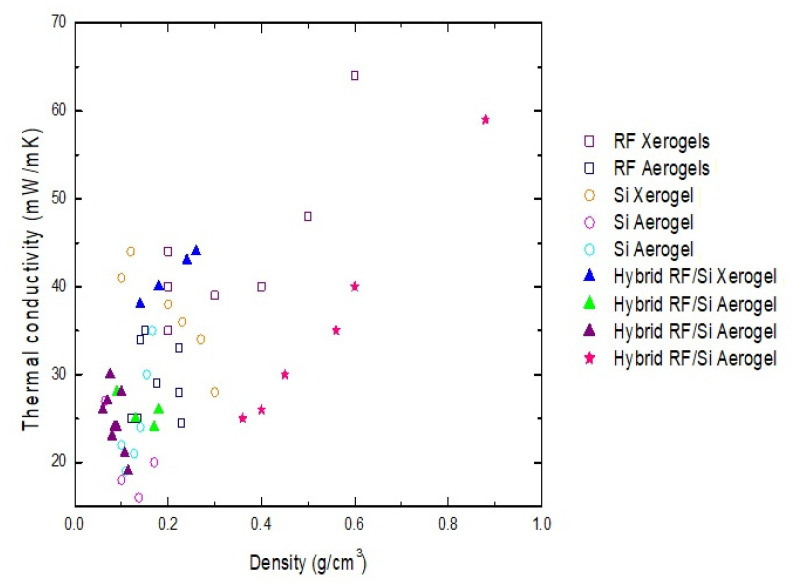
Comparison of insulation performance of the hybrid materials developed in this work, with hybrids, RF and Si gels from other references.

Analyzing the behavior of the hybrid xerogels through the different heat transfer methods is useful for understanding their insulating performance. In the case of the hybrid xerogels synthesized in this study, the contribution of the solid network had a significant impact on the reduction in the total thermal conductivity. Such contribution is related to the tortuosity factor, property which can be modulated by modifying the synthesis conditions [32]. This information provides a new perspective for the optimization of insulating properties from underexplored variables (i.e., solid parameters). Moreover, the hybrid series synthesized in this work had thermal conductivities ranging between 25 and 60 mW/mK, which position them as good insulating materials (see Figure 7). These values are similar or better than those obtained for other hybrids described in the literature. Brilliantova et al. [20] and Berthon et al. [21] prepared hybrid RF/Si aerogels with similar thermal conductivities (ca. 25 mW/mK) (see these references in Figure 7). However, their low-density values were due to the use of supercritical drying, a non-viable practice for large-scale production. On the other hand, by comparison with non-hybrid gels (Si and RF) from References [10,31] dried under ambient pressure, the advantage of the RF/Si hybrid xerogels for a better insulation performance is clear (see Figure 7). Moreover, the synthesis process used in this study is fast and cheaper, due to a 66% reduction in required synthesis time and the elimination of supercritical drying or solvent exchange steps used with other technologies. Therefore, from the simple process described here, materials with a competitive insulation performance were obtained. 

## 4. Conclusions

RF/Si hybrid xerogels had a higher insulating performance than the RF and Si non-hybrid xerogels synthesized in this work. This result is a combination of their chemical composition, porous properties and microstructure. Such characteristics had a specific contribution to the thermal conductivity of the samples and were related to the different heat transfer methods (radiation, gas conductivity and solid conductivity). Radiation was highly related to the chemical composition of the xerogels and decreased with the Si content. The gas conductivity of the samples was considerably reduced with the pore size and envelope density. Finally, the solid conductivity was minimized when the microstructure presented higher roughness and tortuosity, and this contribution was the one with the higher impact on the thermal conductivity of the studied samples. Therefore, by modulating all these properties, hybrid xerogels with 10–15% of Si content, a pore size around 97 nm, porosities higher than 70% and envelope densities below 0.40 g/cm^3^ showed the lowest thermal conductivities (i.e., 25 and 26 mW/mK). Moreover, improvements in insulating materials could be achieved without the use of special and costly drying processes as those employed with aerogels. Based on the correlations discussed in this work, competitive synthesis methods focused on the optimization of hybrid materials can be taken into account for the future of insulating gels. This work can be considered as a gateway to this new approach since the synthesized materials were sufficiently attractive to compete with those obtained from more expensive or complex methodologies reported previously.

## Figures and Tables

**Table 1:** Measurement conditions and thermal properties obtained for reference samples.

Reference	Inputs		Results *		
	Power Heat	Analysis Time	Thermal Conductivity	Thermal Diffusivity	Specific Heat
	W	S	mW/mK	mm^2^/s	MJ/m^3^ K
Foamglass^®^	0.02	160	42.7 (42)	0.43 (0.42)	0.10 (0.10)
CASSPIR^®^	0.02	160	22.2 (22)	0.28	0.08
Lumira^®^	0.01	640	19.9 (21)	0.12 (0.12)	0.17 (0.18 ^a^)

* Thermal specifications of these commercial samples within the brackets. ^a^ Experimental value from reference [12].

**Table 2:** Porous characteristics of xerogels employed in this study.

Sample	*ρ_He_*	*ρ_env_*	*P*	*V_T_*	*d_p_*	S_BET_
	(g/cm^3^)	(g/cm^3^)	%	(cm^3^/g)	(nm)	(m^2^/g)
Si-0.50	2.05	0.50	76	1.54	38	568
Si-0.70	2.08	0.69	67	0.98	18	543
RF-0.45	1.42	0.44	69	1.55	44	107
RF-0.55	1.43	0.54	62	1.16	44	110
RF/Si-0.35-C	1.46	0.36	76	2.11	102	76
RF/Si-0.40	1.43	0.40	72	1.91	92	83
RF/Si-0.45-C	1.47	0.45	69	1.59	42	216
RF/Si-0.45	1.46	0.46	68	1.35	35	128
RF/Si-0.55-C	1.41	0.56	60	1.12	43	110
RF/Si-0.55	1.45	0.57	61	1.14	31	111
RF/Si-0.60-C	1.47	0.60	59	1.01	25	229
RF/Si-0.85	1.56	0.88	43	0.48	28	75
RF/Si-0.50–70%	1.68	0.48	71	1.48	5395	ND

## Supplementary Materials

The following supporting information can be downloaded at: https://www.mdpi.com/article/10.3390/ma15010265/s1, Table S1: Synthesis conditions used for hybrid samples; Table S2: Synthesis conditions used for non-hybrid samples; Figure S1: Isotherms and pore size distribution of xerogels samples; title; Table S3: Thermal conductivity of hybrid xerogels.
