# Supplementary material for: Hybrid RF-Si Xerogels: A Cost-Effective Proposal for Insulator Materials
Source: Materials (Basel). 2021 Dec 30;15(1):265. doi: 10.3390/ma15010265 (PMC8745971; doi:10.3390/ma15010265)
Supplement: Supplementary file 1 [file materials-15-00265-s001.zip › materials-1528858-supplementary.pdf]

Hybrid RF-Si xerogels: a cost-effective proposal for insulator materials

Samantha L. Flores-López[1], Sara F. Villanueva[1], Natalia Rey-Raap[2]*, Ana Arenillas[1]*

[1]Instituto de Ciencia y Tecnología del Carbono, INCAR-CSIC. Francisco Pintado Fe, 26. 33011 Oviedo, Spain

[2]Department of Physical and Analytical Chemistry, Oviedo University-CINN-CSIC, 33006-Oviedo, Spain

*corresponding authors: aapuente@incar.csic.es¸ nataliarey@uniovi.es

Supplementary Data

S1. Synthesis conditions for hybrid materials

Table S1. Synthesis conditions used for hybrid samples.

Sample	Molar ratios			
	R/TEOS	R/F	EtOH/TEOS	D
RF/Si-0.35-C	3	0.5	0	12.5
RF/Si-0.40	6	0.3	0	7.4
RF/Si-0.45-C	3	0.3	0	7.1
RF/Si-0.45	3	0.3	0	12.9
RF/Si-0.55-C	3	0.5	5	12.5
RF/Si-0.55	3	0.3	0	7.1
RF/Si-0.60-C	3	0.13	0	5.9
RF/Si-0.85	3	0.5	5	12.5
RF/Si-0.50-70%	0.1	0.5	4.7	5.6

S2. Synthesis of non-hybrid materials

Si xerogels were synthesized from the reagent concentrations in Table S1. TEOS, ethanol and water were mixed by adding them in this order while keeping magnetic stirring. The pH was adjusted with an ammonia solution 2M. Once the solution was homogeneous, 20mL of NH_4 were added to favor the gelation. Samples were sealed and heated under MW radiation at 60ºC for 2h to form the alcolgel. Then, 250ml of EtOH were added and the sealed sample was maintained at 40ºC in the MW oven for 4h to complete the curing step. Finally, the samples were dried at 30ºC until constant weight.

Two different RF xerogels were prepared with the synthesis conditions shown in Table S1. The precursor solution was prepared by dissolving R in the quantity of water calculated from the D ratio. Then the corresponding amount of F was added, and the mixture was maintained in magnetic agitation to homogenize. The pH of the solution was adjusted with NaOH 1M to achieve a pH of 4.5. The synthesis process was performed under MW heating at 85ºC for a total of 5h to ensure gelation and aging. Then the sample was completely dried in an electrical oven at 60ºC overnight.

Table S2. Synthesis conditions used for non-hybrid samples.

Material	Molar Ratios		pH	MeOH Content (%)
	R/F	D		
RF 45	0.13	6.2	4.5	10.0
RF 55	0.60	4.6	4.5	12.5
Material	Molar Ratios		pH	
	W/TEOS	EtOH/TEOS		
Si 50	6	6	10	
Si 70	2	6	10	

S3. Porous and thermal properties of synthesized xerogels

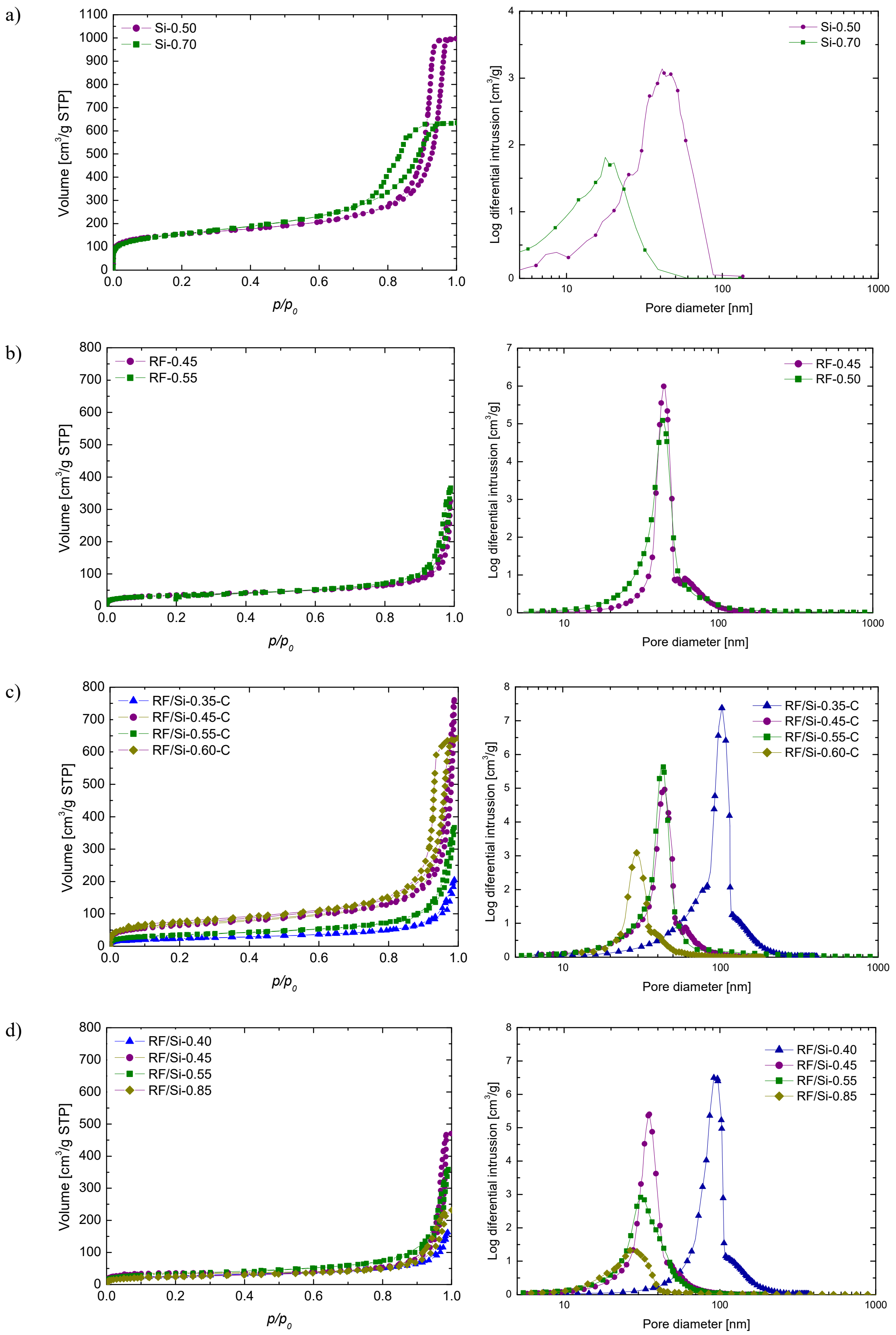

Figure S1. Isotherms and pore size distribution of xerogels samples.

Table S3. Thermal conductivity of hybrid xerogels.

Sample	Thermal conductivity	
	**1-2mm** mW/mK	**0.212-0.5mm** mW/mK
RF/Si-0.35-C	30	25
RF/Si-0.40	30	26
RF/Si-0.45-C	32	30
RF/Si-0.45	-	30
RF/Si-0.55-C	-	36
RF/Si-0.55	41	35
RF/Si-0.60-C	45	40
RF/Si-0.85	66	59
RF/Si-0.50-70%	-	34
